# One-Stage Surgical Management of an Asymptomatic Maxillary Sinus Mucocele with Immediate Lateral Sinus Lift and Simultaneous Implant Placement: A Case Report

**DOI:** 10.3390/jcm14061946

**Published:** 2025-03-13

**Authors:** Alexandru Burcea, Claudia Florina Bogdan-Andreescu, Cristina-Crenguţa Albu, Cristian-Viorel Poalelungi, Andreea-Mariana Bănățeanu, Emin Cadar, Liviu Gabriel Mirea, Laurenţiu-Camil Bohîltea

**Affiliations:** 1 Department of Speciality Disciplines, “Titu Maiorescu” University, 031593 Bucharest, Romania; 2 Helpdent Dental Clinic, 030175 Bucharest, Romania; 3 Department of Genetics, Faculty of Dentistry, “Carol Davila” University of Medicine and Pharmacy, 020021 Bucharest, Romania; 4 Department 9, Faculty of Medicine, “Carol Davila” University of Medicine and Pharmacy, 020021 Bucharest, Romania; 5 Faculty of Pharmacy, “Ovidius” University, 900470 Constanta, Romania; 6 Department of Medical Genetics, Faculty of Medicine, “Carol Davila” University of Medicine and Pharmacy, 020021 Bucharest, Romania

**Keywords:** maxillary sinus, cone-beam computed tomography, mucocele, oral surgery, sinus floor augmentation, platelet-rich fibrin, xenograft, dental implants

## Abstract

**Background:** The relationship between dental implants and sinus mucoceles is an area of growing interest in oral and maxillofacial surgery, as therapeutic approaches for these conditions remain controversial. This case report presents a 48-year-old male with no significant medical history who sought dental care due to recurrent abscesses on the distal abutment of a five-unit maxillary bridge. Clinical and radiographic evaluations, including Cone-Beam Computed Tomography (CBCT), revealed a pathologic lesion associated with the second molar, insufficient alveolar bone height in the posterior maxilla, and a radiopaque mass on the sinus floor. **Methods:** A one-stage surgical approach was planned, involving the second molar extraction, the sinus cyst removal, sinus floor elevation, and simultaneous implant placement. The prosthetic restoration was completed six months postoperatively. **Results:** Over an 88-month follow-up period, no prosthetic complications were observed, and the patient reported high satisfaction with the restoration’s function and aesthetics. **Conclusions:** This case highlights a single-stage surgical strategy’s feasibility and long-term success in managing asymptomatic maxillary sinus mucoceles while optimizing implant placement and rehabilitation.

## 1. Introduction

Maxillary sinus augmentation has long been recognized as a reliable and effective technique for bone augmentation in the posterior maxilla [1,2,3]. Two primary approaches are utilized for sinus floor elevation: the lateral (external) sinus lift and the crestal (internal) sinus lift. The lateral approach is more invasive but provides better visibility and allows for a more significant bone height gain [4]. In contrast, the crestal approach is less invasive and is recommended when the residual bone height exceeds 6 mm, with an expected increase of 3–4 mm [4].

One of the significant challenges in sinus augmentation is the presence of maxillary sinus pathology, particularly sinus cysts, which can complicate the surgical approach and impact the long-term success of implant placement [5,6]. These cystic lesions may compromise sinus membrane integrity, reduce available bone height, and increase the risk of complications such as membrane perforation, sinusitis, or implant failure [7]. Proper identification and classification of sinus cysts are essential to optimize treatment planning and ensure successful sinus augmentation.

Maxillary sinus cysts are classified into three main types: antral pseudocysts, mucous retention cysts, and true sinus mucoceles [8]. Antral pseudocysts are inflammatory exudates that accumulate between the antral mucosa and underlying bone without an epithelial lining [8]. In contrast, mucous retention cysts and mucoceles are both lined with epithelium. Mucous retention cysts result from the obstruction and subsequent dilation of seromucous gland ducts. In comparison, mucoceles are expansile, potentially destructive lesions that, if untreated, can erode and perforate sinus walls [9]. Recognizing and differentiating these cystic lesions is essential for appropriate surgical planning and the successful placement of dental implants.

A sinus mucocele is a benign, epithelial-lined cystic lesion filled with mucus that develops within the paranasal sinuses due to obstruction of the sinus ostium [10,11]. Paranasal mucoceles account for approximately 2–10% of all mucoceles [12], and their formation may be induced by inflammation, infection, or trauma [13]. These lesions are expansile and can cause various symptoms depending on their size and extension into adjacent structures, including facial pain, headaches, and nasal congestion [14].

Although sinus mucoceles are uncommon, they have significant clinical implications when they develop. They can lead to progressive sinus expansion, bone remodeling, and compression of nearby structures [15]. Due to their slow-growing but potentially aggressive nature, mucoceles can contribute to bone resorption, which may compromise the available bone height necessary for sinus augmentation and implant placement [16]. When mucoceles are located near the maxillary sinus floor, they may obstruct sinus drainage and interfere with sinus lift procedures.

Despite being benign, mucoceles require vigilant monitoring and routine follow-up to prevent potential complications [17]. In many cases, maxillary sinus mucoceles remain undiagnosed until they are incidentally detected during radiological examinations for dental implant planning. Their removal may be necessary in cases where they are symptomatic or contribute to infection-related complications that could lead to grafting failure [18,19,20,21].

Surgical management of a sinus mucocele before sinus floor elevation should be considered when its presence affects sinus ventilation or compromises the success of bone grafting procedures [22]. Small cystic masses typically do not impact the prognosis of sinus bone grafting, as demonstrated in a retrospective study involving 23 subjects [23]. Some studies suggest sinus floor augmentation can be performed without complications if the distance between the top of the mucocele and the osteomeatal complex is at least 22 mm or if the mucocele is not exceeding 18 mm in diameter [24]. These parameters help ensure that lifting the sinus membrane does not obstruct the antral drainage duct [24].

The lateral approach is generally favored for mucocele removal during sinus floor augmentation, as it provides optimal surgical site visualization, reduces complications, and facilitates safe membrane detachment. For improved access to the sinus cavity, many authors advocate for a wide flap that fully exposes the lateral maxillary sinus wall, combined with a large bone window, to simplify manipulation and enhance surgical outcomes [25]. A wide approach is recommended in narrow sinuses, as it ensures safe and efficient loosening and reflection of the sinus membrane [2]. A small osteotomy is associated with surgical difficulties and better surgical skills, but reduced postoperative pain and edema [25]. While there is no strict definition distinguishing a small from a large lateral sinus lift, osteotomy size is often categorized as follows: small lateral sinus lift: ~6 × 6 mm and large lateral sinus lift: ~10 × 6 mm [25].

As a final point, maxillary sinus floor elevation is a well-documented technique for restoring adequate bone volume in the posterior maxilla and supporting the placement of dental implants [20], but the relationship between dental implants and sinus mucoceles is an area of growing interest in oral and maxillofacial surgery because therapeutic strategies for these clinical situations remain controversial in the literature.

This study aims to present a case of one-stage surgical management of an asymptomatic maxillary sinus mucocele, demonstrating the feasibility and effectiveness of simultaneous mucocele removal, lateral sinus floor augmentation, and immediate implant placement.

## 2. Case Presentation

### 2.1. Clinical History

A 48-year-old male patient from an urban area with no significant medical history was referred to the Helpdent Dental Clinique due to recurrent abscess formation at the distal abutment of a five-unit maxillary bridge. The patient is a non-smoker, consumes alcohol occasionally, and maintains good oral hygiene. His occupation requires frequent travel by car, and he occasionally experiences back pain. He is not on any chronic medication.

During the anamnesis, the patient reported experiencing abscess formation at the last maxillary right molar for several years, which was managed with intermittent antibiotic therapy. Previous dental radiographs revealed the poor condition of the abutment, which had a hopeless prognosis. Due to the molar abutment’s inadequate dental and periodontal support, his current dentist recommended replacing the existing bridge with an implant-supported restoration.

Various treatment options, including no treatment, a shortened dental arch, and a removable partial denture, were discussed with the patient. The patient chose the implant-supported restoration to restore esthetics and mastication. A Cone-Beam Computed Tomography (CBCT) scan was indicated to thoroughly evaluate the surgical site and determine the optimal treatment approach.

While conventional radiographs provide only two-dimensional images with potential distortions and overlapping structures, CBCT offers high-resolution, three-dimensional imaging, allowing for precise anatomical assessment. Compared to medical computer tomography, CBCT provides superior visualization of bone and dental structures with significantly lower radiation exposure, making it the preferred imaging modality for implant planning and surgical guidance [26,27].

### 2.2. Imaging and Treatment Planning

A CBCT scan provides a comprehensive pre-surgical assessment, evaluating the available bone volume and the adjacent anatomical structures, such as the maxillary sinus. The CBCT findings determine each patient’s anatomical and individual parameters, allowing the selection of an appropriate surgical approach.

**Figure 1:**
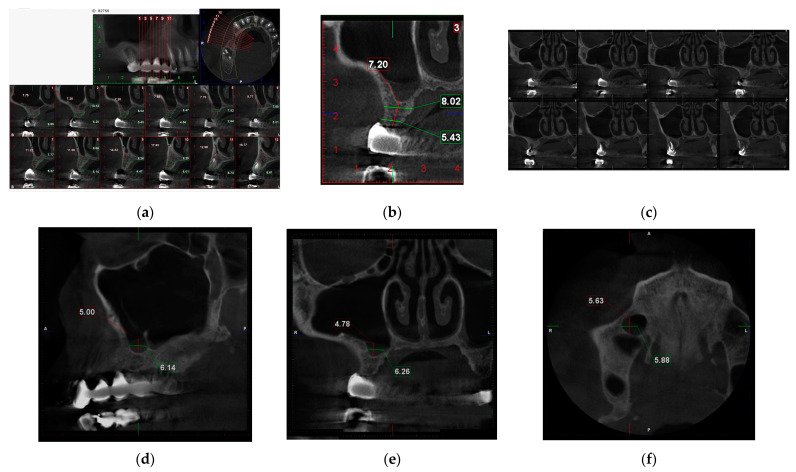
(**a**) Details showing radiolucency around the mesial root of the second molar, bone height in the edentulous area, and sinus septa; (**b**) a faintly radiopaque lesion on sinus floor, with fluid-like density content and 7.20 mm bone height in the first molar area; (**c**) there are no signs of mucosal swelling, and the maxillary ostium is open; (**d**) mediosagittal view of faintly radiopaque lesion on sinus floor and sinus septa; (**e**) frontal and (**f**) transversal view of faintly radiopaque lesion.

In this case, the CBCT scan revealed significant findings, including pathology affecting the second molar, insufficient alveolar bone height for implant placement in the posterior maxilla, a well-defined faintly radiopaque lesion on the sinus floor, and a sinus septa in the molar area (Figure 1a). The available bone height varied, measuring 14 mm at the first premolar site, 9 mm at the second premolar site, and 7 mm at the first molar site.

**Figure 2:**
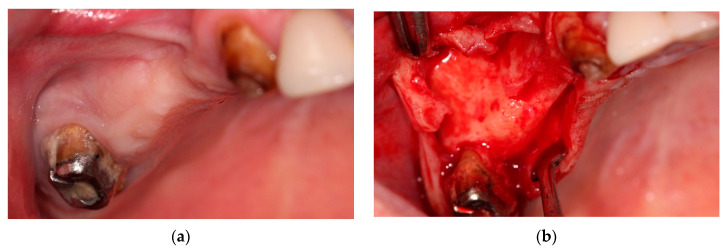

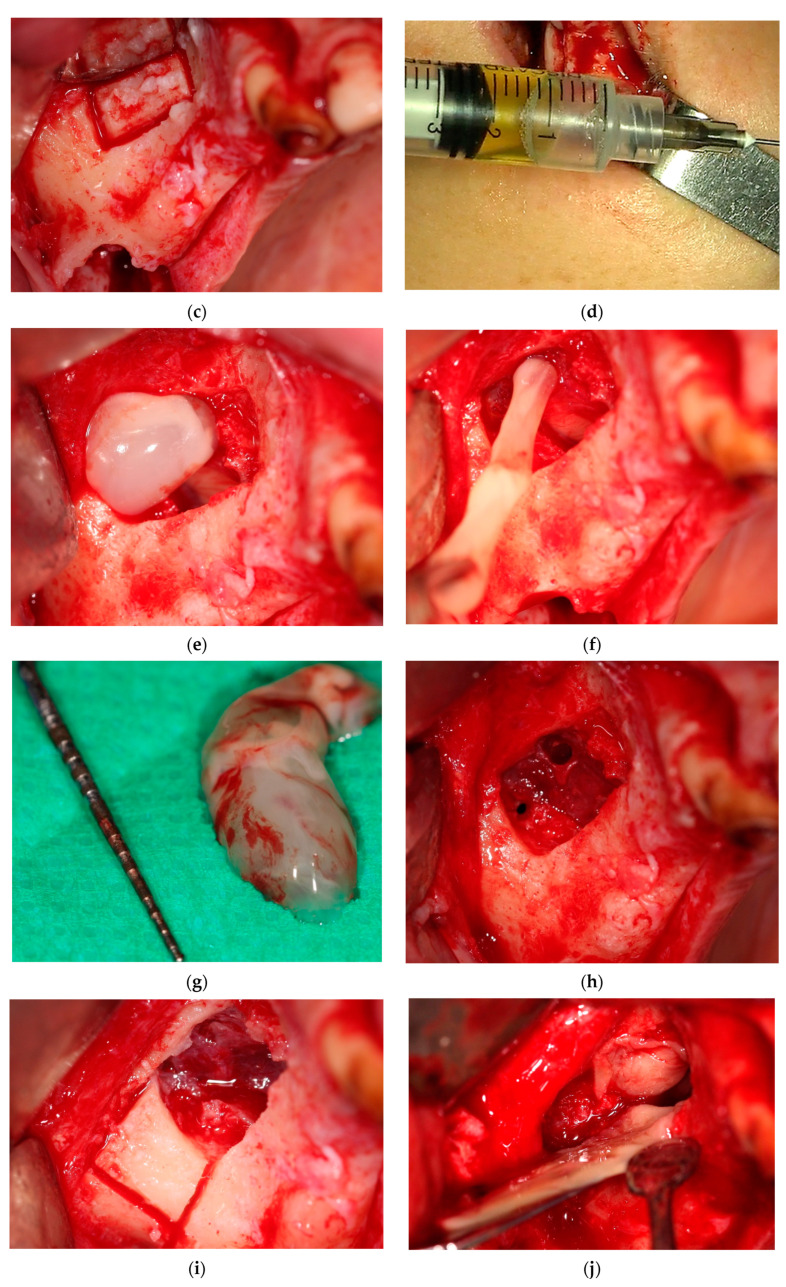

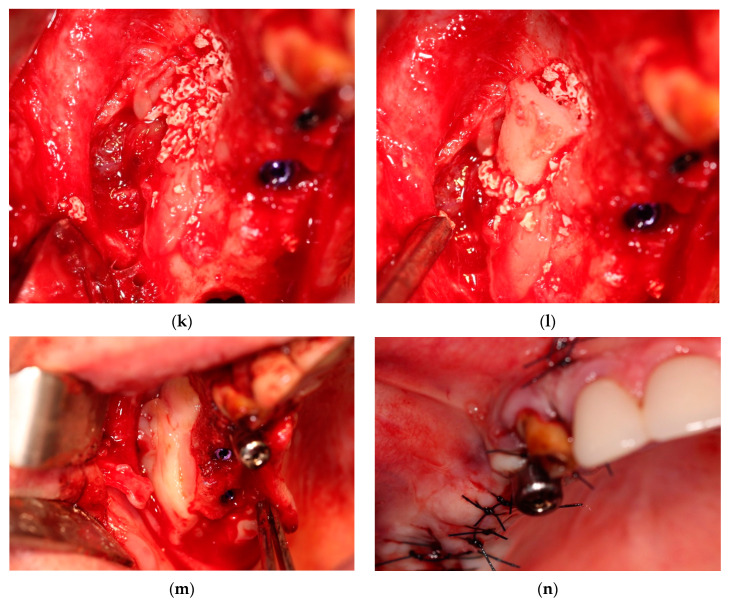
(**a**) Initial situation; (**b**) trapezoidal shape incision and raising the flap; (**c**) the bony lid design and the extraction of the molar; (**d**) the yellow liquid content of the cyst; (**e**) deflation of the mucocele; (**f**) removing of the deflate mucocele; (**g**) the mucocele; (**h**) the aspect of the sinus mucosa after removing the mucocele with two perforations; (**i**) extending the bony lid; (**j**) PRF on top of the sinus membrane to cover the two small perforations; (**k**) the bone graft set in place and implants; (**l**) covering the graft with the bony lids; (**m**) PRF on top of the lid; (**n**) the suture with Supramid 4/0, RC, 3/8, 19.

The well-defined faintly radiopaque lesion on the sinus floor has a flat shape and homogenous content and is located in the region of the first molar and second premolar. Its maximum height and width are in the first molar area (Figure 1b). The location of the lesion coincides with the area of reduced bone height, where augmentation would be necessary to place an implant of at least 10 mm in length, which is considered a standard implant [28]. Notably, the CBCT scan did not indicate any signs of sinusitis or obstruction in the maxillary sinuses (Figure 2c). The size of the faintly radiopaque lesion varies according to the reference plane, with a diameter of around 6 mm (Figure 2d–f).

Based on the CBCT evaluation and a thorough discussion with the patient, the following treatment plan was established: a one-stage surgical procedure involving the extraction of the second molar, removal of the sinus mucocele, sinus floor elevation, and simultaneous implant placement.

One treatment alternative considered was the use of short implants, which offer advantages in terms of cost and efficiency. This approach reduces surgical time and postoperative complications by avoiding the need for bone augmentation. However, the long-term success of short implants remains controversial, particularly in the maxilla, where failure rates tend to be higher than in the mandible [29]. Additionally, to ensure biomechanical stability, an increased number of short implants would be required to distribute occlusal forces and reduce stress on the surrounding bone [30]. Given these considerations, along with the patient’s age, sex, and anatomical parameters, standard-length implants were selected as the preferred option.

The sinus mucocele removal was deemed necessary due to its expansile behavior and proximity to the augmented area. Leaving it in place would risk the success of the bone graft and implant integration.

The decision to proceed with a one-stage surgical approach was based on several factors. Simultaneous implant placement reduces the overall treatment duration, minimizing the number of surgical interventions and postoperative recovery periods—an important consideration given the patient’s frequent travel commitments. Furthermore, CBCT findings indicated that primary implant stability could be achieved, as the minimum available bone height was 7 mm, making immediate placement possible. Addressing the sinus pathology in the same surgical session prevents additional surgical morbidity and simplifies the healing process. Alternative approaches, such as a two-stage surgery with delayed implant placement following bone augmentation, were discussed but were not preferred due to the extended treatment timeline and the patient’s preference for a more efficient solution.

Before proceeding, the patient underwent a comprehensive informed consent process. The proposed treatment plan was thoroughly explained, including its benefits, risks, and potential complications. Key concerns such as sinus membrane perforation, infection risk, and implant failure rates were discussed in detail. The patient was also informed of alternative treatment options, including short implants, staged approaches, or removable prostheses. After addressing all questions and ensuring the patient’s complete understanding, written informed consent was obtained for the treatment plan and the surgical procedure.

### 2.3. Surgical Technique

#### 2.3.1. Preoperative Preparation

The patient was prescribed a preoperative regimen consisting of Cefuroxime 500 mg (Zinnat, GlaxoSmithKline, London, UK) and Metronidazole 500 mg (Metronidazol, Arena Group, Voluntari, Romania). The regimen consists of taking one tablet every 12 h, starting 24 h before surgery and continuing for seven days.

Before administering local anesthesia, the patient performed preoperative gargling with a 0.2% chlorhexidine solution to reduce the bacterial load in the oral cavity. Local anesthesia was provided using Articaine with Epinephrine 1:200,000 (Ubistesin^TM^, 3M Espe, Neuss, Germany), administered via a posterior superior alveolar block and a greater palatine block.

#### 2.3.2. Surgical Steps

The edentulous area exhibited well-keratinized mucosa, which is favorable for future prosthetic reconstruction (Figure 2a). A trapezoidal incision was made to ensure adequate surgical access, creating a bone window extending from the canine’s mesial surface to the second molar’s distal surface. A mucoperiosteal flap was then elevated to expose the surgical site fully (Figure 2b).

Following flap elevation, the second molar was extracted with minimal trauma to preserve the surrounding bone. After extraction, a sinus window, approximately 8 × 6 mm, was created using a piezotome (Mectron Piezosurgery^®^, Carasco, Italy) (Figure 2c) at the level of the second premolar and first molar, where the mucocele was located. The bony lid was carefully detached and stored in a saline solution.

The Schneiderian membrane was gently punctured near the first molar at its estimated lowest point to access the mucocele. A 22-gauge needle attached to a 5 mL syringe was inserted through the Schneiderian membrane to aspirate the yellowish cystic fluid (Figure 2d), reducing the mucocele’s size and creating a small perforation. The cystic membrane was then exposed and carefully removed through a second perforation at the center of the osteotomy (Figure 2e,f). A fine-tip surgical suction was used to facilitate the removal of the cyst, which herniated through the bony window and was clamped using fine anatomical tweezers—the enucleated mucocele measured approximately 10 mm in length (Figure 2g).

Following cyst removal, two small perforations were present in the Schneiderian membrane:

- A 3 mm perforation from the aspiration of the cystic fluid, located along the lateral part of the sinus window, approximately <3 mm from the sinus wall.
- A 6 mm perforation from the cyst removal is situated along the superior border of the bony window.

These were classified as Class IIb and Class I perforations according to the Fugazzoto and Vlassis classification [31]. The first perforation required further osteotomy to expose an intact section of the sinus membrane beyond the perforated area. Consequently, the bone window was extended to allow further elevation of the sinus membrane in the distal and posterior directions (Figure 2i).

The Schneiderian membrane was carefully detached and elevated. The perforations were repaired using a platelet-rich fibrin (PRF) membrane obtained from the patient’s venous blood sample following Choukroun’s protocol [32], using a PRF Duo Quattro centrifuge (Figure 2j). The PRF membrane covered the defects, allowing for simultaneous sinus augmentation. PRF was selected to repair sinus membrane perforation because it provides a biocompatible scaffold rich in growth factors, promoting soft tissue healing and angiogenesis [33,34]. Its fibrin matrix enhances the stability of the sinus membrane repair, improving wound healing [35] and implant stability [36].

The next step involved bone grafting and implant placement. Implant sites in the first and second premolars, and the first molar regions were prepared. The space between the Schneiderian membrane and the sinus floor was filled with a mixture of Endobone^®^ xenograft granules (Zimmer Biomed, Warsaw, Indiana) and shredded PRF cloths, combined with liquid PRF and liquid Metronidazole (Braun Melsungen AG, Melsungen, Germany) to optimize bone regeneration [37,38]. Xenograft granules (Endobone^®^) provide long-term volumetric stability after sinus lift and have a high success rate, particularly when residual bone height exceeds 4 mm, making them suitable for immediate implant placement in sinus augmentation cases [39,40]. Shredded PRF cloths enhance osteogenic potential and accelerate soft tissue healing, and liquid PRF makes the grafting mixture more cohesive and easier to handle. Liquid Metronidazole was included to control perioperative anaerobic contamination, reducing the risk of sinus infections and postoperative complications [41].

Following successful sinus membrane elevation and grafting, three standard-length implants (10 mm length, 4.5 mm diameter) were placed (Figure 2k):

- First premolar region: the implant was placed in the pristine bone, where the bone height was 14 mm. A 10 mm implant was chosen despite the available height due to the presence of a sinus septum, which could impact implant angulation and stability.
- Second premolar and first molar regions: Implants were placed in the augmented bone, where pre-graft residual height was 7–9 mm.

All implants were inserted with a torque of 35 Ncm, ensuring adequate primary stability for immediate healing. Stability was confirmed by tactile feedback during insertion, and the engagement of implants within both native and grafted bone was assessed.

The osteotomy sites were covered with bony lids retrieved during window creation (Figure 2l) and further protected with a PRF membrane (Figure 2m) to aid healing and prevent graft migration. A PRF clot was placed in the extraction socket to facilitate soft tissue regeneration.

A healing abutment was positioned on the implant and placed in the pristine bone to allow for a temporary restoration, reducing prosthetic loading on the augmented sites.

The surgical site was closed with Supramid^®^ (B. Braun, Germany) sutures (Figure 2n), ensuring tension-free wound closure for optimal postoperative healing.

Figure 2 presents details of surgical technique.

#### 2.3.3. Postoperative Care

The patient was prescribed Nimesulide 100 mg (Aulin, Angelini Pharma, București, Romania) every 12 h for pain management and inflammation control.

The patient was advised to follow these specific guidelines to ensure optimal healing and minimize complications:

1. Cold therapy for edema and hematoma prevention. Apply ice packs to the affected cheek the day of surgery and the following day. Use 15 min application intervals, followed by 30 min breaks, to reduce swelling. Avoid direct pressure on the area—wrap the ice pack in a clean towel. The sinus lift and mucocele removal can cause significant facial swelling and cooling the area helps minimize postoperative edema, hematoma formation, and discomfort.
2. Dietary restrictions to avoid pressure on the graft and implants. Consume soft, room-temperature foods on the day of surgery. Continue a soft diet until suture removal. Avoid hard, sticky, or hot foods that could dislodge the graft material, disrupt healing, or exert pressure on the implants. The bone graft and implant sites require stability for osseointegration and chewing on the affected side could compromise graft consolidation and implant stability.
3. Sleeping position for reducing sinus pressure and swelling. Avoid direct cheek contact with the pillow for the first two nights. Keep the head elevated using two pillows to prevent excessive swelling. Elevating the head reduces sinus congestion, prevents excessive blood flow to the surgical site, and minimizes swelling that could increase postoperative discomfort.
4. Oral hygiene for infection prevention. To keep the area clean, rinse gently with a lukewarm saline solution (3 times/day for 10 days). Avoid brushing the suture area, but maintain regular hygiene for the rest of the mouth. Proper hygiene is critical for preventing infection, particularly after sinus surgery, where bacterial contamination can lead to sinusitis or graft failure.
5. Physical activity restrictions to prevent sinus pressure dislodging of the graft. Avoid strenuous activities such as lifting heavy objects, bending forward, or vigorous exercise until sutures are removed. Increased blood pressure and physical strain can cause bleeding, graft displacement, or sinus membrane complications.
6. Pain management for controlling post-surgical discomfort. Postoperative pain is expected to be moderate and should be controlled with prescribed analgesics. Pain may result from bone augmentation, sinus lifting, and soft tissue manipulation and should be managed to reduce stress-induced inflammation.
7. Swelling and edema are common post-surgical effects. Swelling is normal, usually peaking on days 2–3 after surgery. Sinus elevation and mucocele removal involve soft tissue dissection and bone manipulation, increasing localized inflammation. Swelling should gradually subside within a week.
8. Sinus precautions for protecting the sinus membrane and graft stability. Avoid sneezing with a closed mouth—if necessary, sneeze with an open mouth to reduce pressure. Do not blow your nose forcefully; if necessary, do so gently. Avoid coughing forcefully for 2–4 weeks. Increased sinus pressure from sneezing, nose blowing, or coughing could dislodge the sinus graft, cause membrane perforation, or lead to sinus infection.
9. Minor bleeding from the nose after sinus surgery. Slight postoperative bleeding from the nostril on the affected side is normal. This occurs due to sinus mucosal manipulation and osteotomy during the sinus lift. However, persistent or heavy bleeding should be reported immediately.
10. Avoid manipulating the surgical site to prevent implant failure and graft disruption. Do not press, massage, or apply pressure to the operated area. Rationale: Any external force could disrupt the sinus graft, implant integration, or wound healing.

These postoperative care instructions are specifically tailored to address the risks associated with sinus augmentation, mucocele removal, and simultaneous implant placement, ensuring optimal healing, reduced complications, and implant success.

### 2.4. Follow-Up

The patient was monitored through scheduled follow-ups at one week, one month, three months, and six months postoperatively, followed by annual assessments up to 94 months. These evaluations focused on healing progression, implant success, and patient satisfaction.

**Figure 3:**
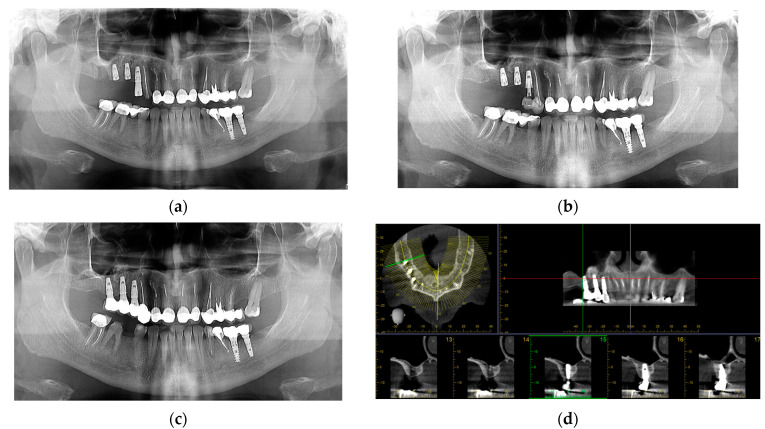
(**a**) Orthopantomogram immediately postoperative in March 2017; (**b**) orthopantomogram 6 months later, with temporary crowns; (**c**) orthopantomogram 62 months later, there were no postoperative complications; (**d**) CBCT 94 months later without sinus pathology.

An orthopantomogram was taken immediately following the intervention (Figure 3a) and a temporary prosthesis was performed for esthetic reason on canine and first implant with the help of digital technology [42,43], while the atrophic maxilla remains unloaded during the healing interval for the graft consolidation [44] (Figure 3b).

During the one-week follow-up, pain levels, soft tissue healing, swelling, infection signs, and suture integrity were assessed. The patient reported mild discomfort that was well managed with prescribed analgesics. No signs of infection, wound dehiscence, or excessive swelling were observed.

At the one- and three-month follow-up, soft tissue adaptation and the absence of sinus-related complications (e.g., nasal congestion, sinusitis) were evaluated. The healing process was uneventful, without pain or inflammation.

At the six-month follow-up, the definitive prosthetic restoration was done. All implants exhibited good stability, with no mobility, discomfort, or inflammation. The orthopantomogram showed adequate marginal bone levels and no peri-implant radiolucency. After verifying occlusal harmony and prosthetic fit, the definitive three-unit screw-retained bridge was placed (Figure 3c).

During long-term follow-up (annual assessments for 94 months), there were no signs of implant failure (mobility, pain, suppuration, or excessive bone loss); marginal bone loss remained within acceptable limits, less than 1 mm in the first year [45], stable thereafter. No recurrence of mucocele or sinus-related complications was noticed (Figure 3d).

Success was determined using standard implant success criteria, ensuring the following [46]:

Absence of persistent pain or discomfort, confirmed in all follow-ups;

- No implant mobility, confirmed via clinical testing;
- No peri-implant bleeding or suppuration, soft tissues remained healthy;
- No radiolucent lesions on CBCT/X-rays, bone integration was stable;
- Marginal bone loss less than 1 mm in the first year, confirmed on radiographs.

The long-term stability of the implant–prosthetic complex was successfully demonstrated over 94 months without biological or mechanical complications.

A structured interview was conducted at six months and annually thereafter to assess the patient’s satisfaction with both functional and esthetic outcomes.

At the six-month follow-up, the patient rated chewing efficiency, speech, and overall comfort on a scale from 1 to 10 (1 = very dissatisfied, 10 = completely satisfied) to 8/10.

The patient reported aesthetic and functional satisfaction 10/10 at the final prosthesis placement and maintained this rating in subsequent follow-ups regarding prosthesis appearance, alignment, function, and integration with natural dentition.

As regards the overall experience and expectations regarding postoperative recovery, pain perception, and impact on daily activities, the patient strongly preferred the one-stage approach due to its reduced treatment time and minimal surgical interventions.

This case highlights a clinically successful and patient-satisfactory outcome following one-stage sinus lift, mucocele removal, and implant placement. The implants remained stable over 94 months of follow-up, the sinus augmentation procedure proved effective, and the patient maintained full functional and esthetic satisfaction.

## 3. Discussion

### 3.1. Summary of Key Findings

The key findings in this case report are as follows: a single-stage approach, management of sinus mucocele in context of implant surgery, the use of PRF for repairing Schneiderian membrane perforations and sinus floor augmentation, and long-term follow-up on the stability of sinus grafts and implants placed after mucocele removal.

While staged approaches are often recommended in case of sinus pathology to prevent complications, this demonstrates that a carefully planned one-stage approach can be safe and effective in patients with an asymptomatic mucocele and sufficient residual bone height.

This approach reduces treatment time and the need for multiple surgical interventions, enhancing patient recovery and satisfaction.

This case provides clinical evidence supporting simultaneous mucocele removal, sinus lift, and implant placement in one-stage surgery.

PRF membranes help to repair Schneiderian membrane perforations, which appear after mucocele removal. They also facilitate better graft integration and enhanced soft tissue healing.

Most studies report short-to-mid-term follow-ups for sinus lifts in the relationship of sinus cysts. Still, this case provides long-term evidence (nearly 8 years) of implant and graft stability post-mucocele removal.

The absence of implant failure, graft resorption, or mucocele recurrence underlines the reliability of this surgical protocol.

### 3.2. Comparison with Existing Literature

A healthy sinus environment is essential for the success of sinus floor elevation, reducing the risk of postoperative complications even in cases of minor perforation [20]. Preoperative evaluation should identify otorhinolaryngological contraindications that might affect sinus physiology and address them before surgery [20].

There is no consensus on the optimal sinus augmentation approach for patients with maxillary sinus mucoceles. Different strategies have been proposed depending on the type and size of the cyst [5]:

- Spontaneous drainage for pseudocysts;
- Aspiration and delayed augmentation for mucous retention cysts smaller than 20 mm;
- Cyst enucleation with delayed augmentation for cysts larger than 20 mm to ensure sinus membrane recovery.

Similarly, for antral pseudocysts, studies suggest the following [47]: endoscopic surgery concurrent with augmentation, sinus augmentation without biopsy, sinus augmentation with pseudocyst removal. However, no standardized protocol exists for choosing among these options.

For maxillary mucoceles, treatment generally ranges from conservative management (monitoring or aspiration) to surgical removal. Marsupialization has been associated with high recurrence rates [48], whereas complete removal significantly lowers recurrence [49]. The ear-nose-throat (ENT) specialist removes the mucocele using endoscopic intranasal sinus surgery, often under general anesthesia. Endoscopic surgery boasts a low recurrence rate, shorter postoperative recovery time, and reduced morbidity [12,50,51,52]. During the procedure, the surgeon removes the medial wall of the maxillary sinus while accessing the inferior nasal meatus [53] and a bone defect remains in the medial wall. While endoscopic removal via an ENT specialist is often preferred due to low recurrence rates and minimal morbidity, it does not permit simultaneous sinus augmentation, requiring an additional surgical stage.

It is important to remember that mucoceles have osteolytic properties and invasive behavior. For this reason, it is advisable to remove them when a sinus lift is needed.

No consensus exists on whether sinus floor elevation should be performed immediately after mucocele removal or in a staged approach. Several studies propose different protocols; Nosaka et al. [21] advocate a two-stage approach, suggesting that the cystic contents may interfere with graft healing, bone augmentation could be compromised if residual cystic material remains, and a minimum of four months should be allowed for sinus healing before augmentation. Jadach et al. [54] introduce the Croco Eye technique, which prioritizes immediate sinus augmentation while minimizing Schneiderian membrane perforation.

Testori et al. [20] reported successful augmentation after aspiration of cystic contents with a 5-year follow-up, while Hadar et al. [49] showed that complete removal is preferred over marsupialization due to a lower recurrence rate, which aligns with the surgical approach.

Given the absence of sinusitis symptoms, patency of the ostium, and adequate residual bone height (≥7 mm), a simultaneous approach was chosen, combining the following:

- Lateral sinus wall access, which allows direct visibility for mucocele removal, ensures proper sinus membrane elevation, and facilitates bone grafting and implant placement;
- Schneiderian membrane perforation repaired with PRF provides predictable healing, enabling immediate augmentation without increased risk;
- Implant placement in a single surgical session reduces treatment time, patient morbidity, and the need for multiple interventions, aligning with Croco Eye technique principles [54].

While some authors advocate for staging augmentation after cyst removal [21], studies show that simultaneous sinus lift and mucocele removal are feasible in well-selected cases [20]. This approach is particularly advantageous when the sinus membrane can be successfully repaired intraoperatively, such as in this case, using PRF membranes, there is sufficient bone height, over 4 mm for primary implant stability, and the procedure can be performed under local anesthesia, avoiding the need for general anesthesia and additional surgeries.

However, it is essential to acknowledge the potential risks of this technique, including graft contamination, membrane perforation, and limited long-term data in the context of simultaneous sinus lift and mucocele removal.

### 3.3. Explanation of Unique Aspects of the Case

This case presents a one-stage surgical approach for managing an asymptomatic maxillary sinus mucocele. It combines simultaneous mucocele enucleation, lateral sinus lift, and immediate implant placement. The approach is noteworthy because it demonstrates a successful long-term outcome with a 94-month follow-up. This case adds to the growing evidence supporting one-stage surgical protocols for sinus augmentation in the presence of asymptomatic mucoceles. It demonstrates that proper case selection, meticulous surgical technique, and biologically active materials, such as PRF, can optimize outcomes, reducing surgical interventions and total treatment time while ensuring long-term implant success.

### 3.4. Clinical Implications and Recommendations

Pre-surgical analysis of each sinus for bone augmentation and choosing the correct surgical approach is mandatory to avoid intra-operative and postoperative complications [55,56]. CBCT images of the maxillary sinus are a prerequisite for evaluating the maxillary sinus in patients undergoing sinus floor elevation. Therefore, CBCT is essential in diagnosing mucoceles, which appear as well-defined, faintly radiopaque lesions. They are mainly observed as dome-shaped lesions on the maxillary sinus floor.

CBCT provides detailed, three-dimensional imaging of the maxillary sinus, allowing for precise localization and measurement of mucoceles [57]. Unlike orthopantomogram, CBCT enables better differentiation between mucoceles, retention cysts, and other sinus pathologies based on their shape, location, and density [58]. CBCT helps evaluate cortical bone thinning, expansion, or resorption caused by the mucocele, which is critical when planning sinus augmentation [59].

While CBCT provides excellent bony detail, it lacks the ability to differentiate soft tissue characteristics, making it challenging to distinguish mucoceles from other cystic lesions purely based on imaging. CBCT does not provide functional information about sinus ventilation or drainage, which can be critical in determining the risk of postoperative complications.

Due to these limitations, CBCT should always be complemented by intraoperative findings such as mucus aspiration and histopathological analysis to confirm the diagnosis before proceeding with sinus augmentation.

The most common causes of mucoceles are chronic infection, allergic sinonasal disease, trauma, and previous surgery, but the cause remains uncertain in 64% of the patients in a retrospective study [50]. Histopathological examination demonstrates a membrane made up of pseudostratified ciliated cylindrical epithelium (sinus mucosa) supported by fibrovascular connective tissue, which contains mucus without evidence of neoplastic pathology [60].

In the clinical management of sinus mucoceles, understanding the role of mucus viscosity is critical, significantly influenced by genetic mutations in MUC5AC and MUC5B genes [61] and exacerbated by inflammatory cytokines like IL-6, IL-13, and TNF-α [62,63]. These cytokines not only promote mucin overproduction, leading to increased viscosity and impaired mucociliary clearance [64,65], but also engage in pathogenic processes such as osteoclastic bone resorption, particularly mediated by IL-1 and TNF-alpha, which are critical in the formation of mucoceles by increasing mucus viscosity through heightened glycosylation and excessive mucin secretion [66]. This interaction between cytokine activity and mucin genes underscores the challenges in surgically treating mucoceles, as it affects the sinus structure directly and can complicate the removal of thickened mucus [67,68,69]. Tailoring surgical and preoperative strategies to these molecular and genetic insights can lead to improved management outcomes, focusing on reducing mucus viscosity and mitigating its osteolytic effects.

In this case report, two small perforations were intentionally created: a minor one for the aspiration of the mucocele and a larger one for its removal. A PRF membrane was chosen to cover the sinus perforations, as it promotes the regeneration of damaged tissues and speeds up the healing process [38,70]. Beyond its healing advantages, the PRF membrane is easily manipulated and well suited for the surgical area. Additionally, another PRF membrane was applied to cover the bone graft, helping to hold the particulate bone together while acting as a barrier to facilitate new bone formation [71,72,73].

When repairing Schneiderian membrane perforations, PRF offers a minimally invasive and biologically active method that enhances healing, reduces complications, and improves surgical outcomes [74,75]. In this case, PRF was used to seal two intentional perforations covering the sinus graft to stabilize the particulate bone and promote osteointegration, and mix with xenograft to accelerate healing.

PRF effectively repairs the membrane because it is rich in growth factors, including platelet-derived growth factor (PDGF), which stimulates fibroblast proliferation and extracellular matrix production; transforming growth factor-beta (TGF-β), which encourages epithelial cell proliferation and collagen synthesis; vascular endothelial growth factor (VEGF), which promotes angiogenesis essential for delivering oxygen and nutrients to the repair site; and epidermal growth factor (EGF), which aids in epithelialization and wound closure [76,77,78].

In this case, the approach is on the lateral sinus wall, allowing good visibility, mucocele removal, Schneiderian membrane elevation, controlled bone graft, and implant placement when the residual bone height is above 4 mm [79]. Moreover, crestal sinus lift is not recommended in the presence of a cyst, sinus septa, or bone height below 6 mm [80]. A CBCT scan before surgery is mandatory to assess not only the pathology of the sinus but also different anatomical variations, such as the presence of the septa, its relationship with the roots of remaining teeth, the presence of blood vessels, alteration in the mucosa, and variations in cavity size [81,82].

Since implant treatment can be considered successful if the implants function uneventfully for more than 5 years after treatment when combined with maxillary sinus floor elevation [83], in this case, with a 94-month follow-up after surgery, we could consider the surgical intervention and prosthetic reconstruction a success. Despite the favorable outcomes in implant survival, maintenance of augmented bone height, no recurrence of cystic lesions, and long-term observation period, the findings are limited in small-sized samples. Further research is necessary to establish standardized guidelines for managing sinus mucoceles in implant dentistry.

This case documents a clinically successful outcome with long-term stability and high patient satisfaction, providing valuable insights for clinicians managing sinus pathologies during implant surgery. It emphasizes the importance of CBCT-driven diagnosis, careful surgical execution, and PRF-based membrane repair in achieving predictable outcomes in complex cases.

## 4. Limitations

While this case demonstrates a successful one-stage approach for managing an asymptomatic maxillary sinus mucocele with simultaneous sinus augmentation and implant placement, several limitations must be acknowledged.

This is a single-case report, and while the outcome was favorable, broader conclusions cannot be drawn without larger clinical studies or randomized controlled trials.

Patient-specific factors, such as sinus anatomy, immune response, and healing capacity, may have contributed to this case’s success, making it difficult to generalize these findings to all patients with sinus pathology.

The procedure was performed by an experienced surgeon, which may not reflect outcomes in less experienced hands.

Surgical variations, such as sinus lift techniques, alternative membrane repair methods, or grafting materials, could influence clinical outcomes.

## 5. Conclusions

This case report presents a rare instance of asymptomatic maxillary sinus mucocele managed through a one-stage surgical approach, integrating mucocele removal, lateral sinus floor augmentation, and simultaneous implant placement. While sinus lift procedures and implant placement are well documented, the concurrent management of a sinus mucocele within the same surgical session is less frequently reported.

The single-stage approach minimized surgical interventions, reducing total treatment time and postoperative morbidity, which is particularly beneficial for patients with time constraints.

Maxillary sinus mucoceles are often incidental findings on CBCT, and their management in implant-driven treatment plans is not well established.

This case demonstrates how careful CBCT evaluation and surgical planning can allow for safe, simultaneous removal and augmentation without compromising implant success rates.

With a nearly 8-year follow-up, this case provides rare long-term data on the stability of sinus grafts and implants placed after mucocele removal, reinforcing the feasibility of this combined surgical approach.

## Data Availability

The original contributions presented in the study are included in the article, further inquiries can be directed to the corresponding authors.
